# An Application of a Hybrid MCDM Method for the Evaluation of Entrepreneurial Intensity among the SMEs: A Case Study

**DOI:** 10.1155/2014/703650

**Published:** 2014-08-10

**Authors:** Reza Rostamzadeh, Kamariah Ismail, Hossein Bodaghi Khajeh Noubar

**Affiliations:** ^1^Department of Management, Universiti Teknologi Malaysia, 81310 Skudai, Johor, Malaysia; ^2^Department of Management, East Azarbaijan Science & Research Branch, Islamic Azad University, Tabriz, Iran

## Abstract

This study presents one of the first attempts to focus on critical success factors influencing the entrepreneurial intensity of Malaysian small and medium sized enterprises (SMEs) as they attempt to expand internationally. The aim of this paper is to evaluate and prioritize the entrepreneurial intensity among the SMEs using multicriteria decision (MCDM) techniques. In this research FAHP is used for finding the weights of criteria and subcriteria. Then for the final ranking of the companies, VIKOR (in Serbian: VlseKriterijumska Optimizacija I Kompromisno Resenje) method was used. Also, as an additional tool, TOPSIS technique, is used to see the differences of two methods applied over the same data. 5 main criteria and 14 subcriteria were developed and implemented in the real-world cases. As the results showed, two ranking methods provided different ranking. Furthermore, the final findings of the research based on VIKOR and TOPSIS indicated that the firms A3 and A4 received the first rank, respectively. In addition, the firm A4 was known as the most entrepreneurial company. This research has been done in the manufacturing sector, but it could be also extended to the service sector for measurement.

## 1. Introduction

Today, small and medium-sized enterprises (SMEs) are the basis of the global economy and play a vital role in job creation. The vitality and success of SMEs are known as important features in measuring an economy's growth and future development [1]. In developing and emerging economies, SMEs and entrepreneurs play an important role as they represent a major source of employment and generate significant revenue and export earnings [2]. Small firms depend on entrepreneurs to get a firm off the ground. Although SMEs typically face considerable resource constraints, they are often successful innovators [3]. This is mainly because entrepreneurial SMEs are nimbler than their larger counterparts; they can move faster and are more flexible, proactive, and risk keen [4, 5]. As an emerging research field, entrepreneurship has received much attention over the last few decades. However, there is a lack of consensus on what precisely are the critical factors affecting entrepreneurial intense. For example, previous entrepreneurship research has investigated opportunity identification processes [6], startup processes [7] exploitation processes [8], team formation processes [9], financing processes [10], entrepreneurial exits [11], and international entrepreneurship [12, 13]. Yet, little attention has been paid to entrepreneurial intensity (EI). The entrepreneurial intention has been investigated extensively in the West [14–17], though it still remains as an understudied area in Malaysia. Applying the Western studies in Malaysian context would certainly raise a question of their suitability and applicability. At the local setting, some studies are really needed to expand the pertinency and accuracy of the results [18]. This study presents one of the first attempts to focus on critical success factors (CSFs) influencing the EI of Malaysian SMEs as they attempt to expand internationally. A better understanding of the entrepreneurial process would provide an important contribution to the entrepreneurship evaluation literature, and the entrepreneurship intensity literature would benefit from an investigation of how CSFs of entrepreneurship contribute to the SMEs performance. Based on the identified gaps in the literature, the key research questions addressed in this study are as follows: what are the CSFs of the EI? What are the priority of these critical factors? And how are the importances and preferences of these factors affect each of them?

The multicriteria decision making (MCDM) approach is a suitable approach to evaluate critical success factors (CSFs) of EI, as the nature of these evaluation models includes different perspectives and should address the allocation of limited resources. Tzeng and Huang [19] indicated that MCDM is a methodology that can consider multiple criteria at the same time [20] and helps the decision maker to estimate the best case according to the characteristics of limited available cases. The MCDM technique is a powerful tool widely used for evaluating and ranking problems containing multiple, usually conflicting, criteria. It includes several techniques, which allow rating a range of criteria and ranking them as a decision maker. It has great potential to reduce the cost and time and increase the accuracy of decisions and can be an appropriate framework for solving the problems. The concept of combining the fuzzy theory and MCDM is referred to as fuzzy MCDM (FMCDM).

The paper adds to the entrepreneurial evaluation literature in several ways. First, no prior study that we are aware of has examined critical factors of entrepreneurship as a driving force of entrepreneurial intensity. Second, few studies in the literature examine the performance of SMEs regarding this evaluation. The next contribution of this research is that there is no more study using MCDM in fuzzy environment to evaluate EI among the SMEs. This paper is intended to bridge these gaps. The remainder of the paper is as follows. In the next section, we review the existing literature. In Section 3, the methodologies such as FAHP, VIKOR, and TOPSIS are used to assess the criteria. Applications of the proposed methodologies in real-world situations and a comparison of the results are presented in Section 4. The results and discussion are discussed in Section 5. Finally, in Section 6, conclusion and future studies are given.

## 2. Literature Review

In this section, first available MCDM applications in the field of entrepreneurship and innovation are reviewed and then how to evaluate the EI is described.

### 2.1. MCDM Applications in the Field

Han et al. [21] developed an evaluation model based on fuzzy theory and analytic hierarchy process (AHP) for assessing entrepreneurial environment to help the government and entrepreneur in an interacted environment. This model provides a precise, effective, and organized decision support tool. The evaluation of entrepreneurial capacity of college students was done by Ni-Di and Yi [22]. The decision making method has been provided reasonably to pick the satisfactory self-employed student. The outcomes show a greater weight on the dimension of entrepreneurial capacity of college student evaluation, and four critical evaluation criteria related to it are personal characteristics, personal qualities, personal abilities, and environment. Rezaei et al. [23] proposed four different methodologies for measuring the entrepreneurship, including the statistical methodology, a fuzzy logic, a data envelopment analysis (DEA), and a naïve methodology. As an expert-based methodology, fuzzy logic compensates some of the limitations of the statistical methodology. Drawing on a sample of 59 startups, they measured innovativeness, risk taking, and proactiveness and subsequently compared the resulting EO scores. A naïve methodology produces a value that lies between the other results, while the entrepreneurial score from a fuzzy logic methodology is the most dissimilar from the others. Most lately Rezaei et al. [24] applied an improved fuzzy AHP for ranking the firms based on their EO score. Same as their previous work [23], the three dimensions were used: innovativeness, risk taking, and proactiveness. The results specify that proactiveness is the most significant dimension, followed by innovativeness. Also, there are noticeable differences when it comes to the weights of the items. Lu et al. [25] used an AHP and fuzzy set theory, to assess a firm's technological innovation capability by several qualitative and quantitative criteria. Chen et al. [26] provided a quantitative MCDM approach to knowledge management in construction entrepreneurship education by means of an analytic knowledge network process (KANP). The study finds that there are eight clusters and 178 nodes in the KANP. The model and experimental research on the evaluation of teaching cases discloses that the KANP method is effective in conducting a knowledge management of the entrepreneurship education. Čančer and Knez-Riedl [27] presented a method for developing the internal ratings to best select among the business partners of a firm using AHP. Since qualitative factors come into play, special attention is given to determining not only quantitative but also qualitative criteria. Tsai and Kuo [1] applied a decision making trial and evaluation laboratory (DEMATEL), ANP, and zero-one goal programming (GP) methods, to measure entrepreneurship policies, using the Stevenson and Lundstrom [28] proposed criteria such as reducing entry and exit barriers, promotion, entrepreneurship knowledge, and financing and business support.

### 2.2. Entrepreneurial Intensity

**Figure 1:**
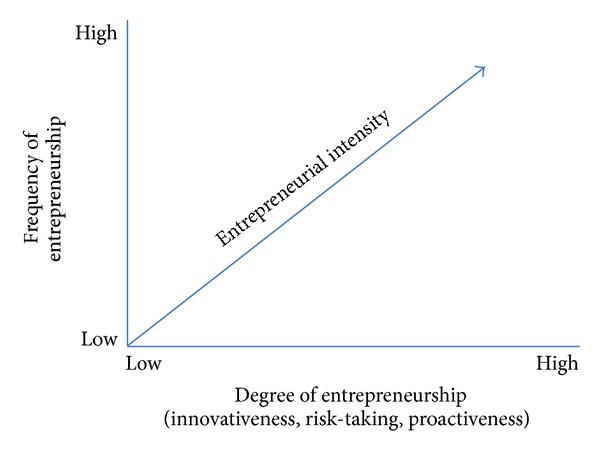
An illustration of entrepreneurial intensity.

In entrepreneurship research, entrepreneurial orientation (EO) has been found to have a positive impact on firm performance [29–35] and to be one of the most popular branches within entrepreneurship research [36]. Firms with high levels of EO tend to constantly scan and monitor their operating environment in order to find new opportunities and strengthen their competitive positions [37]. To define entrepreneurship, many authors [38–40] believe that entrepreneurship can be described as “the process of creating value by bringing together a unique combination of resources to exploit an opportunity.” This definition exposes that the entrepreneurship may vary in terms of extent and the number of times it occurs. Several dimensions of EO have been proposed in which entrepreneurial intensity has been highlighted as the most important one. Miller [41] appears to offer the earliest operationalization of the EO concept and propose three-dimension proactiveness, risk taking, and innovativeness. Sexton and Morris [30] mention the varying levels of entrepreneurship as entrepreneurial intensity (EI). They view EI as a function of the degree and frequency of entrepreneurship as shown in Figure 1 [30]. This is supported by Antoncic and Hisrich [42]. On the other hand, Lumpkin and Dess [43] claimed that not three but five dimensions should be used to measure entrepreneurship, namely, autonomy, competitive aggressive, proactiveness, innovativeness, and risk taking. Also, Yang et al. [44] reviewed the literature of corporate entrepreneurship (CE) and explored the relationship between CE and market performance in China by taking a disaggregated approach after developing a reliable and valid scale of CE suggested by Antoncic and Hisrich [42]. They used venturing, innovation, self-renewal, proactiveness, and market performance as an evaluation scale. The results showed that each dimension exerts differentiated impacts on market performance of firms.

Frequency of entrepreneurship refers to the number of times an enterprise acts entrepreneurially while the degree of entrepreneurship is measured by three subdimensions: innovativeness, risk taking, and proactiveness. Kraus [45] investigated the role of the EO in service firms in Austria. A significant positive relationship between EO and corporate performance could be identified, with a clear emphasis on innovative behavior as the most important subdimension. Rigtering et al. [46] studied a comparative analysis of the EO/growth relationship with service and manufacturing firms using Miller's [41] dimensions. The findings showed that service firms have a significantly higher EO than manufacturing firms, both on the overall level and for each of the three dimensions. Scheepers et al. [47] compared the e-business EI of the Johannesburg Securities Exchange (JSE) and ICT companies. Company characteristics, organizational factors, and environmental factors were compared. It has been confirmed that EI varies among different industries or company groups. Erasmus and Scheepers [48] studied the relationship between EI and shareholder value creation. An adapted CE measurement instrument is applied in order to gauge EI, while shareholder value creation is measured by the market adjusted total share return (TSR) and the value based financial performance measure economic value added (EVA). The contribution of the study is the focus on the relationship between EI and shareholder value creation, rather than purely on the accounting-based financial performance of an enterprise. Keh et al. [35] studied the effects of EO and marketing information on the performance of SMEs among Singaporean. The results indicate that EO plays an influential role in the acquisition and utilization of marketing information and also has a direct effect on firm performance. The utilization of information regarding marketing mix decisions (particularly the promotion and place elements) positively affects firm performance, and it partially mediates the relationship between EO and firm performance. Related to entrepreneurial intentions, Lee et al. [49] investigated the influence of organizational and individual factors in Singapore. They examined why individuals intend to leave their jobs to start business ventures. Findings suggest that work environments with an unfavorable innovation climate and/or lack of technical excellence incentives influence entrepreneurial intentions, through low job satisfaction. Cardon et al. [50] measured entrepreneurial passion (EP) and provided conceptual foundations and scale validation. They developed and validated an instrument to capture EP and its inherent dimensions. The results indicate that proper measurement of entrepreneurial passion incorporates the interaction between entrepreneurs' feelings and identity centrality for each domain.

The first subdimension, specifically innovativeness, refers to the creation of new products, services, and technologies. The second subdimension, risk taking, involves the willingness to commit significant resources to opportunities with a reasonable chance of costly failure. These risks are typically calculated and manageable. The third subdimension, proactiveness, reflects the top management orientation to pursuing enhanced competitiveness and includes initiative, competitive aggressiveness and boldness [51]. The forth subdimension, autonomy, refers to the independent action of an individual or a team in bringing forth an idea or a vision and carrying it through to completion. Generally, autonomy means the ability and will to be self-directed in the pursuit of opportunities.The fifth subdimension, competitive aggressiveness, refers to a firm's propensity to directly and intensely challenge its competitors to achieve entry or improve the position; EO literature agrees that a competitive aggressive orientation is one of the basic characteristics of successful entrepreneurial firm activity (e.g., [29, 43]).

All the above-mentioned five dimensions have been used infrequently in the EO literature when compared with the use of the model with three dimensions. Rauch et al. [52] stated that only in one study [53] all these five dimensions have been used. However, in 30 studies [54–58], these same three dimensions have been used. In this research, we applied Lumpkin and Dess's dimensions and developed subcriteria for evaluating of EI in fuzzy environment.

## 3. Fuzzy Set Theory

### 3.1. FAHP

The Analytic hierarchy process (AHP) introduced by Saaty [59] directs how to determine the priority of a set of alternatives and the relative importance of attributes in a MCDM problems. Yang and Chen [60] stated that the classic AHP has some deficiencies. It is useful only when data are crispy, dealing with a very unbalanced scale of judgment; uncertainty of human judgment does not take into account of natural language; provided ranking is rather imprecise; and the subjective judgment of perception, evaluation, improvement, and selection based on preference of decision makers has great influence on the AHP results. To overcome such vagueness, ambiguity, and subjectivity of the human judgment process, fuzzy set theory has been introduced [61]. Decision makers express their opinions in terms of linguistic scales. Linguistic data are converted into fuzzy numbers with the help of different membership functions. Then, it becomes easy to solve MCDM problems. Therefore, fuzzy set theory has become a helpful tool for systematizing human activities with uncertainty-based information. As Ragin [62] stated, most scholars have not been familiar with the potential of fuzzy logic for transforming social science methodologies. Although it has great capability for dealing with vagueness problems in the field of innovation and entrepreneurship as a social science; yet we find a few applications in the existing studies.

**Figure 2:**
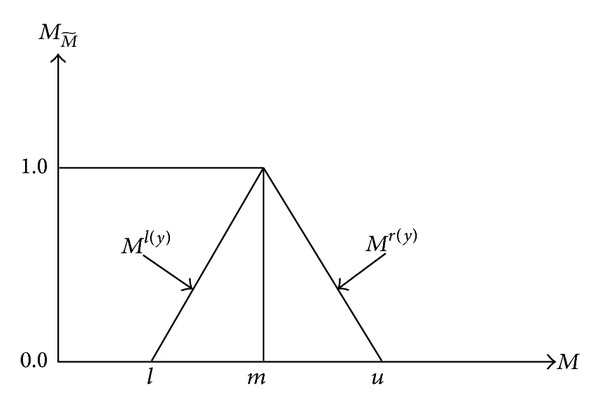
Triangular fuzzy number $\tilde{M}$.

A tilde “~” will be placed above a symbol if the symbol represents a fuzzy set. A triangular fuzzy number (TFN) $\tilde{M}$ is shown in Figure 2. A TFN is denoted simply as (*l*∣*m*,  *m*∣*u*) or (*l*, *m*, *u*). The parameter *l* denotes the smallest possible value, the parameter *m* the most promising value, and the parameter *u* the largest possible value. Each TFN has linear representations on its left and right side such that its membership function can be defined as

(1) µ(x ∣ M~)={0,x<l,x−lm−l,l≤x≤m,u−xu−m,m≤x≤u,0,x>u.

A fuzzy number can always be given by its corresponding left and right representation of each degree of membership:

(2) M~=Ml(y),  Mr(y)=(l+(m−l)y,u+(m−u)y),y∈[0,1].

There are many fuzzy AHP methods proposed by various authors [63–69]. The FAHP methodology is presented in the Appendix. The weights that are obtained from fuzzy AHP are considered and used in VIKOR and TOPSIS calculations.

### 3.2. VIKOR

Recently, the VIKOR method has been introduced as an applicable technique to implement within MCDM. Opricovic [70] developed VIKOR (VlseKriterijumska Optimizacija I Kompromisno Resenje) for multicriteria optimization of complex systems. This method determines the compromise solution and is able to establish the stability of decision performance by replacing the compromise solution obtained with initial weights. VIKOR can be divided into the following steps [70–75] starting from *L*_*p*_-metric used as an aggregating function in a compromise programming method [76, 77]:

(3) Lpi={∑j=1n[wj(fj∗−fij)fj∗−fj−  ]p}1/p,1≤p≤+∞; i=1,2,…I.  

In the VIKOR method, (*L*_1,*i*_ as *S*_*i*_) and (*L*_*∞*,*i*_ as *R*_*i*_) are used to formulate ranking measure. The solution obtained by min⁡ *S*_*i*_ is with a maximum group utility (“majority” rule), and the solution obtained by min⁡ *R*_*i*_ is with a minimum individual regret of the “opponent.” The algorithm of the VIKOR method has the following steps.

**Step 1 .**

Determine the best *f*_j_* and the worst *f*_*j*_^−^ values of all attribute functions, *j* = 1,2,…, *n*. If the *j*th function represents a benefit, then we have

(4) $$\begin{array}{c} f_{j}^{*}=\max\left(f_{ij}\right),\;\;f_{j}^{-}=\min\left(f_{ij}\right), \end{array}$$

where *f*_j_* is the positive ideal solution for the *j*th criteria and *f*_*j*_^−^ is the negative ideal solution for the *j*th criteria. If one associates all *f*_j_*, one will have the optimal combination, which gets the highest scores, the same as *f*_*j*_^−^.

**Step 2 .**

Compute the values *S*_*i*_ and *R*_*i*_,  *i* = 1,2,…, *m* by

(5) $$\begin{array}{c} S_{i}=\overset{n}{\underset{j=1}{\sum }}\left[\frac{w_{j}\left(f_{j}^{*}-f_{ij}\right)}{f_{j}^{*}-f_{j}^{-}}\;\right],\\ R_{i}=\underset{j}{\max}\left[\frac{w_{j}\left(f_{j}^{*}-f_{ij}\right)}{f_{j}^{*}-f_{j}^{-}}\right], \end{array}$$

where *S*_*i*_ denotes the distance rate of the *i*th alternative to the positive ideal solution and *R*_*i*_ represents the distance rate of the *i*th alternative to the negative ideal solution. Also *w*_*j*_ are the weights of criteria, expressing their relative importance.

**Step 3 .**

Compute the values *Q*_*i*_, *i* = 1,2,…, *m* by

(6) $$\begin{array}{c} Q_{i}=v\frac{S_{i}-S^{*}}{S^{-}{-S}^{*}}+\left(1-v\right)\frac{R_{i}-R^{*}}{R^{-}{-R}^{*}}, \end{array}$$

where *S* ^−^ = max⁡_*i*_ *S*_*i*_, S* = min⁡_*i*_ *S*_*i*_, *R* ^−^ = max⁡_*i*_ *R*_*i*_, R* = min⁡_*i*_ *R*_*i*_, and *v* is the weight of the strategy of “the majority of criteria” (or “the maximum group utility”); here suppose that *v* = 0.5.

**Step 4 (rank the alternatives).**

According to the *Q*_*i*_ values calculated by Step 3, we can rank the alternatives to make decision.

**Step 5 .**

If the following two conditions are satisfied concurrently, then the scheme with a minimum value of *Q* in ranking is considered the optimal compromise solution, such that

 (C1) the alternative *Q* (*A* ^(1)^) has an acceptable advantage, if *Q* (*A* ^(2)^) − *Q* (*A* ^(1)^) ≥ 1/*n*−1, where *A* ^(2)^ is the alternative with the second position in the ranking list by and *n* is the number of alternatives;
 (C2) the alternative *Q* (*A* ^(1)^) is stable within the decision making process if it is also best ranked in *S*_*i*_ and *R*_*i*_.

**Step 6 .**

Select the best alternative by choosing *Q*(*A* ^(*m*)^) as a best compromise solution with the minimum value of *Q*_*i*_ regarding the above conditions [78, 79].

### 3.3. TOPSIS

Hwang and Yoon [80] originally proposed the order performance technique based on similarity to ideal solution (TOPSIS), in which the chosen alternative should not only have the shortest distance from the positive ideal reference point (PIRP), but also have the longest distance from the negative ideal reference point (NIRP), to solve the MCDM problems.In the following the steps of TOPSIS are given.

**Step 7 .**

Decision matrix is being normalized via

(7) $$\begin{array}{c} r_{ij}=\frac{w_{ij}}{\sqrt{\sum_{j=1}^{J}w_{ij}^{2}}},\;j=1,2,3,\ldots ,J,\;\;i=1,2,3,\ldots ,n. \end{array}$$

**Step 8 .**

The weighted normalized decision matrix is being formed by

(8) $$\begin{array}{c} v_{ij}=w_{i}*r_{ij},\;j=1,2,3,\ldots ,J,\;\;i=1,2,3,\ldots ,n. \end{array}$$

**Step 9 .**

Positive ideal solution (PIS) and negative ideal solution (NIS) will be determined by

(9) $$\begin{array}{c} A^{+}=\left\{v_{1}^{+},v_{2}^{+},v_{3}^{+},\ldots ,n\right\}\;\;\max\;\text{values},\\ A^{-}=\left\{v_{1}^{-},v_{2}^{-},v_{3}^{-},\ldots ,n\right\}\;\;\min\;\text{values}. \end{array}$$

**Step 10 .**

The distance of each alternative from PIS and NIS will be calculated:

(10) di+=∑j=1n(vij−vj+)2, j=1,2,…,J,di−=∑j=1n(vij−vj−)2, j=1,2,…,J.

**Step 11 .**

The closeness coefficient of each alternative will be calculated:

(11) $$\begin{array}{c} {CC}_{i}=\frac{d_{i}^{-}}{{d_{i}^{+}+d}_{i}^{-}},\;i=1,2,\ldots ,J. \end{array}$$

**Step 12 .**

By comparing *CC*_*i*_ values, the ranking of alternatives is determined.

## 4. Implication in Real-World Cases

Malaysia is one of the countries that have an emerging economy. The number of companies is growing quickly and is now becoming a center of new business opportunities as international investors view Malaysia as the place to invest and establish their businesses. Therefore, the entrepreneurship development has become the key agenda which is evident by the introduction of mechanisms that cater to entrepreneurs [81]. In Malaysia, the government has created an enormous amount of funding towards the promotion of entrepreneurship especially for SMEs. The Malaysian government has been extremely encouraging entrepreneurship. Since the 1970s, the government has given due emphasis to increasing Malay ownership and participation in the corporate sector and high-income occupation as outlined in New Economic Policy of 1971 [82]. This objective is further charted and highlighted in the New Development Policy in 1991 through the establishment of Bumiputera Commercial and Industrial Community (BCIC), which is responsible for fostering and developing Malay and other Bumiputera groups as entrepreneurs and professionals [82]. The BCIC has been the main network through which the strengthening of entrepreneurship among the Malays in Malaysia has been encouraged. The establishment of the Ministry of Entrepreneur Development in 1995 clearly indicates the growing importance of the government role in the issue of entrepreneur development [83]. According to the Federation of Malaysian Manufacturer (FMM) directory reports, 34.7 percent of Malaysian SMEs have less than 50 employees, 32.1 percent have 51–150 employees and 33.2 percent of SMEs have more than 150 employees [84]. Hence, in this research, we studied the SMEs with less than 50 employees as they possess the highest percent among the others. In this section, we apply the aforesaid methodology to evaluate the critical factors of EI among the 30 SMEs and rank them based on VIKOR and TOPSIS methods. First data collection process and then the implementation of the methodology are presented.

**Figure 4:**
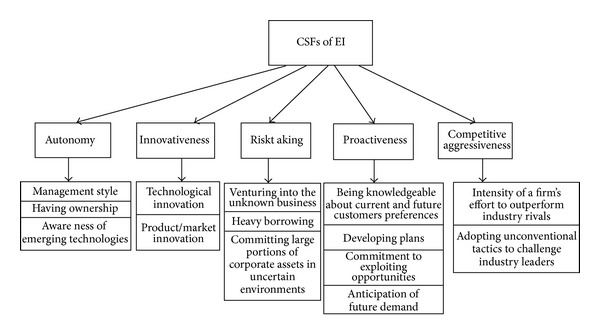
Hierarchy of the problem.

This research has been conducted in three stages. First, FAHP was applied for finding the final weights of main criteria and subcriteria. Next, VIKOR was used to rank the EI of the firms and finally TOPSIS technique was adopted to compare the results with VIKOR. To get the required data of FAHP, a questionnaire was distributed. The respondents of this research were managers, assistant managers, and analysts of companies. To determine the reliability of the questionnaire, Spearman rank correlation coefficient analysis was conducted. Reliability test of the questionnaire was done at 95% confidence coefficient level. As the results show, the questionnaire has acceptable reliability. The hierarchy of the problem can be found in Figure 4, which includes three levels. The top level of the hierarchy represents the ultimate goal of the problem, while the second level of the hierarchy consists of five main criteria, which are, namely, innovativeness, proactiveness, autonomy, competitive aggressiveness, and risk taking. At the third level, these criteria are decomposed into various subcriteria.

Five criteria and fourteen subcriteria are considered, as shown in Table 1. For weighting tables, linguistic scales are used as illustrated in Table 2. Then, the main criteria and subcriteria were calculated using FAHP. Fuzzy pairwise comparisons of the main criteria and subcriteria are given in Tables 3 and 4, respectively. In the end, final weights of criteria and subcriteria using FAHP is presented in Table 5.

### 4.1. Application of VIKOR

In this stage, VIKOR was used to rank the EI of the firms. For this reason, the managers of the company have grouped. Decision makers from different backgrounds may define different weight vectors. They usually cause not only the inexact evaluation but also serious persecution during the decision process. Then, linguistic variables for the criteria and subcriteria weights and also the decision matrix are provided as shown in Tables 6 and 7, respectively. The values for *S*, *R*, and *Q* were calculated and summarized in ascending order as demonstrated in Tables 8 and 9, consequently. Here, the calculation is given for the firm A1. Calculation of the others was done similarly.

**Step 13 .**

Determine the best *f*_j_* and the worst *f*_*j*_^−^ values as (4):

 
*f*_j_*:** 7 8 6 7 7 6 6 7 8 7 7 5 5 8 6 8 7 7 7**
 
*f*_*j*_^−^:* 1 2 3 3 2 2 2 3 3 3 3 1 2 2 2 3 2 2 3*.

**Step 14 .**

Compute the values *S*_*i*_ and *R*_*i*_ by (5):

(12) S1=0.08×7−57−1+0.33×8−78−2+0.1×6−56−3+0.29  ×7−57−3+0.2×7−57−2+0.035×6−36−2+0.005  ×6−56−2+0.039×7−77−3+0.165×8−78−3+0.165  ×7−67−3+0.056×7−57−3+0.025×5−15−1+0.019  ×5−35−2+0.087×8−78−2+0.029×6−36−2+0.087  ×8−68−3+0.087×7−57−2+0.1×7−67−2+0.1×7−77−3=0.026+0.054+0.033+0.145+0.08  +0.026+0.001+0+0.033+0.041+0.028  +0.025+0.012+0.014+0.021+0.034  +0.034+0.02+0=0.63,R1={0.026,0.054,0.033,0.145,0.08,0.026,0.001,0,0.033,0.041,0.028,0.025,0.012,0.014,0.0217,0.034,0.034,0.02,0}=0.145.

**Step 15 .**

Compute the values *Q*_*i*_ by (6):

(13) Q1=0.5×0.63−0.39  1.78−0.39  +(1−0.5)×0.145−0.0720.33−0.072=0.227.

From Table 9, it has been shown that the A3 is best ranked by *Q* and also both (C1) and (C2) conditions are satisfied, meaning that *Q*_A25_ − *Q*_A3_ ≥ 1/30−1 and also A3 is best ranked by *R* and *S*. Therefore, A3 is the best selected company for the best compromise solution.

### 4.2. Application of TOPSIS

In this stage, TOPSIS was conducted for comparing the results with the VIKOR. After normalizing Table 7 via (7), *R* will be obtained:

(14) R=[0.1990.2130.1960.1810.1760.1550.2050.2360.2170.2160.1830.060.160.2320.1390.2010.1920.2040.2290.1590.2430.1570.2170.1760.1030.2050.2030.1860.180.1460.060.1060.2650.1860.2340.1920.170.1640.1990.2130.1960.2170.2110.1550.1640.2030.2480.2520.2560.180.2130.2650.1860.2340.1920.2040.1970.2390.2130.1960.2530.2470.2060.2460.2360.2480.2520.220.120.2670.2320.2320.2680.230.2380.2290.1590.2130.1960.1810.2110.1550.1640.2030.2170.2160.1830.120.1060.1990.2320.2010.1530.2040.1970.1990.2430.2350.2170.2110.1030.1230.1690.1860.180.220.060.160.1990.1860.1670.230.2380.1640.2390.1820.1570.2170.2470.2060.1640.2360.1860.180.2560.120.2670.2320.1860.2010.2680.2040.1640.2780.1820.1960.2170.1760.1550.2460.1690.2480.180.220.240.160.1320.2790.1670.1530.170.1310.1190.1520.1960.1440.2470.310.1230.2030.1550.1440.1460.240.2670.1660.1390.1340.1920.2040.1640.1590.1820.1170.1810.1760.2580.1230.1350.1550.1080.1460.060.2130.1660.1860.2010.1920.2040.2290.1990.1820.1170.1080.1410.1550.0820.1690.1860.180.1830.180.1060.0990.0930.1340.1530.1360.1310.1190.0910.1960.1080.1410.1030.1640.1350.1550.1440.1460.180.1060.0990.1860.1340.1150.0680.1310.0790.0910.1570.1810.1050.1550.2050.1350.0930.1080.110.240.160.1320.0930.1670.0760.1360.1310.0390.060.1170.1440.070.1550.1640.1350.1240.1080.110.120.160.0660.0930.1340.1150.1360.0980.1190.1520.1570.1440.1410.2060.2050.1010.1860.1440.1460.3010.2130.0990.1390.10.1920.170.1640.1590.1520.1570.1080.070.2580.2050.1690.1860.2160.1830.060.160.1320.2320.2010.230.170.1970.1590.2130.1960.2170.210.1550.1640.2030.2170.180.1830.120.1060.1990.2320.2010.1530.170.1970.1990.2130.1960.1440.1760.1550.2050.2360.1860.2160.1830.060.160.1990.1390.2010.1920.2040.2290.1190.2130.1960.2170.1760.1550.1230.2030.2480.2160.2560.180.160.2650.1860.2340.1530.2040.1970.1990.2430.2350.1810.2110.1030.0820.1690.1860.2520.220.060.160.2320.1860.1670.1920.2380.1640.2780.1820.1570.2170.1760.2060.2460.1690.2480.2520.220.3010.2130.1320.2790.1670.1530.170.1640.2780.1820.1170.1440.1410.1550.0820.1690.1550.180.1830.3010.1060.0990.0930.1340.1920.1360.1640.1590.0910.1570.1810.1760.1550.2050.1690.0930.1080.1830.240.160.1320.1390.1670.0760.1360.1970.1990.1520.2350.1080.1410.2060.2460.1010.1860.180.1460.3010.160.1320.1390.10.1920.2380.1640.2390.2430.1960.2170.2470.1550.2050.2030.2170.180.1830.120.160.1990.2320.2010.1920.170.1970.0790.060.1570.1440.1050.1550.1640.1690.1860.1080.110.180.2130.0660.0930.1670.1150.1360.2290.1190.1210.1960.1810.1760.1550.1640.2030.1550.180.1460.180.1060.1320.1860.1340.1150.0680.1640.1590.2130.1960.2170.1760.1550.2050.1690.1860.2160.1830.060.160.2650.1860.2680.1920.170.2290.1990.2130.1570.1810.2470.2060.2050.2360.1860.1440.2560.060.2670.2320.2320.2010.2680.2380.1640.1190.1210.2350.1080.2470.310.2460.1690.1550.1080.1460.3010.2670.1990.1390.1340.230.2040.229].

The weights that we obtained from FAHP were used to get the weighted decision matrix *V*. So, the weighted normalized decision matrix was formed by (8):

(15) V=[0.0160.070.0190.0520.0350.0050.0010.0090.0350.0350.010.0010.0030.020.0040.0170.0160.020.0220.0120.080.0150.0630.0350.0030.0010.0070.030.0290.0080.0010.0020.0230.0050.020.0160.0170.0160.0160.070.0190.0630.0420.0050.0000.0070.040.0570.0140.0040.0040.0230.0050.020.0160.020.0190.0190.070.0190.0730.0490.0070.0010.0090.040.0570.0120.0030.0050.020.0060.0230.020.0230.0220.0120.070.0190.0520.0420.0050.0000.0070.0350.0350.010.0030.0020.0170.0060.0170.0130.020.0190.0160.080.0230.0630.0420.0030.0000.0060.030.0290.0120.0010.0030.0170.0050.0140.020.0230.0160.0190.060.0150.0630.0490.0070.0000.0090.030.0290.0140.0030.0050.020.0050.0170.0230.020.0160.0220.060.0190.0630.0350.0050.0010.0060.040.0290.0120.0060.0030.0110.0080.0140.0130.0170.0130.0090.050.0190.0410.0490.010.0000.0070.0250.0230.0080.0060.0050.0140.0040.0110.0160.020.0160.0120.060.0110.0520.0350.0090.0000.0050.0250.0170.0080.0010.0040.0140.0050.0170.0160.020.0220.0160.060.0110.0310.0280.0050.0000.0060.030.0290.010.0040.0020.0080.0020.0110.0130.0130.0130.0090.030.0190.0310.0280.0030.0000.0050.0250.0230.0080.0040.0020.0080.0050.0110.010.0060.0130.0060.030.0150.0520.0210.0050.0010.0050.0150.0170.0060.0060.0030.0110.0020.0140.0060.0130.0130.0030.0190.0160.0410.0140.0050.0000.0050.0280.0170.0060.0030.0030.0050.0020.0110.010.0130.0090.0090.050.0150.0410.0280.0070.0010.0030.030.0230.0080.0070.0040.0080.0040.0080.0160.0170.0160.0120.050.0150.0310.0140.0090.0010.0060.030.0350.010.0010.0030.0110.0060.0170.020.0170.0190.0120.070.0190.0630.0420.0050.0000.0070.0350.0290.010.0030.0020.0170.0060.0170.0130.0170.0190.0160.070.0190.0410.0350.0050.0010.0090.030.0350.010.0010.0030.0170.0040.0170.0160.020.0220.0090.070.0190.0630.0350.0050.0000.0070.040.0350.0140.0040.0030.0230.0050.020.0130.020.0190.0160.080.0230.0520.0420.0030.0000.0060.030.0570.0120.0010.0030.020.0050.0140.0160.0230.0160.0220.060.0150.0630.0350.0070.0010.0060.040.0570.0120.0070.0040.0110.0080.0140.0130.0170.0160.0220.060.0110.0410.0280.0050.0000.0060.0250.0290.010.0070.0020.0080.0020.0110.0160.0130.0160.0120.030.0150.0520.0350.0050.0010.0060.0150.0170.010.0060.0030.0110.0040.0140.0060.0130.0190.0160.050.0230.0310.0280.0070.0010.0030.030.0290.0080.0070.0030.0110.0040.0080.0160.0230.0160.0190.080.0190.0630.0490.0050.0010.0070.0350.0290.010.0030.0030.0170.0060.0170.0160.0170.0190.0060.0190.0150.0410.0210.0050.0000.0060.030.0170.0060.0040.0040.0050.0020.0140.010.0130.0220.0090.040.0190.0520.0350.0050.0000.0070.0250.0290.0080.0040.0020.0110.0050.0110.010.0060.0160.0120.070.0190.0630.0350.0050.0010.0060.030.0350.010.0010.0030.0230.0050.0230.0160.0170.0220.0160.070.0150.0520.0490.0070.0010.0090.030.0230.0140.0010.0050.020.0060.0170.0230.0230.0160.0090.040.0230.0310.0490.010.0010.0060.0250.0170.0080.0070.0050.0170.0040.0110.020.020.022].

Then positive ideal solution (PIS) and negative ideal solution (NIS) will be determined by (9):

(16) A+={0.022,0.08,0.023,0.073,0.049,0.01,0.001,0.009,0.04,0.057,0.014,0.007,0.005,0.023,0.008,0.023,0.023,0.023,0.022},A−={0.003,0.019,0.011,0.031,0.014,0.003,0,0.003,0.015,0.017,0.006,0.001,0.002,0.005,0.002,0.008,0.006,0.006,0.009}.

The distance of each alternative from PIS and NIS was calculated through (10):

(17) d1+=0.038,  d2+=0.04,  d3+=0.02,d4+=0.013,  d5+=0.039,  d6+=0.036,d7+=0.039,  d8+=0.044,  d9+=0.062,d10+=0.058,  d11+=0.066,  d12+=0.085,d13+=0.084,  d14+=0.095,  d15+=0.067,d16+=0.07,  d17+=0.038,  d18+=0.046,d19+=0.035,  d20+=0.03,  d21+=0.034,d22+=0.06,  d23+=0.078,  d24+=0.067,d25+=0.034,  d26+=0.091,  d27+=0.065,d28+=0.036,  d29+=0.045,  d30+=0.074,d1−=0.072,  d2−=0.08,  d3−=0.089,d4−=0.097,  d5−=0.072,  d6−=0.083,d7−=0.074,  d8−=0.068,  d9−=0.055,d10−=0.059,  d11−=0.05,  d12−=0.025,d13−=0.028,  d14−=0.019,  d15−=0.043,d16−=0.047,  d17−=0.076,  d18−=0.068,d19−=0.077,  d20−=0.089,  d21−=0.079,d22−=0.053,  d23−=0.037,  d24−=0.048,d25−=0.086,  d26−=0.026,  d27−=0.038,d28−=0.075,  d29−=0.076,  d30−=0.052.

The closeness coefficients of each alternative were calculated by (11):

(18) C1+=0.0720.038+0.072=0.654,C2+=0.080.04+0.08=0.66,C3+=0.0890.02+0.089=0.816,C4+=0.0970.013+0.097=0.881,C5+=0.0720.039+0.072=0.648,C6+=0.083  0.036+0.083=0.697,C7+=0.0740.039+0.074=0.655,C8+=0.0680.044+0.068=0.607,C9+=0.0550.062+0.055=0.47,C10+=0.0590.058+0.059=0.504,C11+=0.050.066+0.05=0.431,C12+=0.0250.085+0.025=0.227,C13+=0.0280.084+0.028=0.25,C14+=0.0190.095+0.019=0.166,C15+=0.0430.067+0.043=0.39,C16+=0.0470.07+0.047=0.401,C17+=0.0760.038+0.076=0.67,C18+=0.0680.046+0.068=0.596,C19+=0.0770.035+0.077=0.687,C20+=0.0890.03+0.089=0.747,C21+=0.0790.034+0.079=0.699,C22+=0.0530.06+0.053=0.469,C23+=0.0370.078+0.037=0.321,C24+=0.0480.067+0.048=0.417,C25+=0.0860.034+0.086=0.716,C26+=0.0260.091+0.026=0.222,C27+=0.0380.065+0.038=0.368,C28+=0.0750.036+0.075=0.675,C29+=0.0380.065+0.038=0.368,C30+=0.0520.074+0.052=0.412.

Comparing *CC*_*i*_ values, the ranking of main criteria was determined as follows:

(19) C4>C3>C20>C25>C21>C6>C19>C28>C17>C2>C7>C1>C5>C8>C18>C10>C9>C22>C11>C24>C30>C16>C15>C27>C29>C23>C13>C12>C26>C14.

The summary of ranking based on VIKOR and TOPSIS methods is shown in Table 10.

## 5. Results and Discussions

This paper applied an approach based on the FAHP-VIKOR and FAHP-TOPSIS techniques for evaluating and prioritizing the CSFs of EI among the SMEs. In this research FAHP is used for finding weights of criteria and subcriteria. Then two well-known MCDM techniques for ranking were used together to see the differences of two methods that applied over the same data. Based on obtaining weight by FAHP, innovativeness with 0.33 is placed in first priority, proactiveness with 0.29 in second place, competitive aggressiveness with 0.2 in third place, risk taking with 0.1 in fourth place, and autonomy with 0.08 in fifth place with lower importance. In innovativeness group, technological and product/market innovations earned same weight with 0.5. In proactiveness group, being knowledgeable about current and future customers' preferences, commitment to exploiting opportunities and anticipation of future demand are placed in first priority with 0.087 and developing plans in the second rank with 0.029. In competitive aggressiveness groups, adopting unconventional tactics to challenge industry leaders and intensity of a firm's effort to outperform industry rivals obtained the same weight 0.1. In risk taking group, venturing into the unknown business received the first rank with 0.056, heavy borrowing got the second rank with 0.026, and committing large portions of corporate assets in uncertain environments got the third rank with 0.019. In autonomy group, awareness of emerging technologies with 0.039 received the first rank, management style with 0.035 was in the second, and having ownership got the third rank with 0.005.

The final aggregated EI score of firm *m*, EI_*m*_, is calculated as EI_*k*_ = ∑_*i*=1_^*n*^ *w*_*i*_ *φ*_*im*_, *m* = 1,…*M*, where *w*_*i*_ is the local weight of subcriterion *i*; *φ*_*im*_ is the assigned score to firm *m* with respect to subcriterion *i*; *n* is the number of subcriteria; and *M* is the number of firms. Table 7 shows the final summed scores of the firm. In the last column of Table 7, the relative level of entrepreneurship of the various firms can be found. For example, firm A4, with EI = 0.71, reflected the most entrepreneurial firm, while firm A14, with EI = 0.308, reflects the least entrepreneurial firm.

As depicted in Table 10, three indicators (*Q*, *R*, *S*) in VIKOR method were used to compare the results with the TOPSIS. Comparing TOPSIS with VIKOR (*Q*) values showed that only seven items are compatible. The VIKOR and TOPSIS use different aggregation functions and different normalization methods. The VIKOR method introduces the ranking index based on the particular measure of “closeness” to the ideal solution. In contrast, the basic principle of the TOPSIS method is that the chosen alternative should have the “shortest distance” from the positive ideal solution (PIS) and the “farthest distance” from negative ideal solution (NIS) but it does not consider the relative importance of these distances. These two MCDM methods use different kinds of normalization to eliminate the units of criterion functions: the VIKOR method uses linear normalization, and the TOPSIS method uses vector normalization.

## 6. Conclusion

Previous researches focused on three dimensions of EO (innovativeness, risk taking, and proactiveness); however, to the best of our knowledge, there is no more research that has applied the Lumpkin and Dess five dimensions of entrepreneurship measurement using MCDM techniques. Also, we developed subcriteria for the above-mentioned dimensions of the evaluation. Thus, there is a need for a more structured approach to evaluations and decision making in the field of EI. This research has been done in the manufacturing sector located in Skudai area in Malaysia and only 30 companies have been investigated. For collecting comprehensive data about the situation of entrepreneurial intensity among the Malaysian firms, it would be better to implement in whole country and particularly in the same sector. The proposed criteria of this research could be also extended to the service sector with slight modification based on their needs. In future research, a comparative study using other MCDM methods like PROMETHEE, ELECTRE, and DEMATEL can be applied to assess the EI of firms in fuzzy environment.

## Figures and Tables

**Figure 3:**
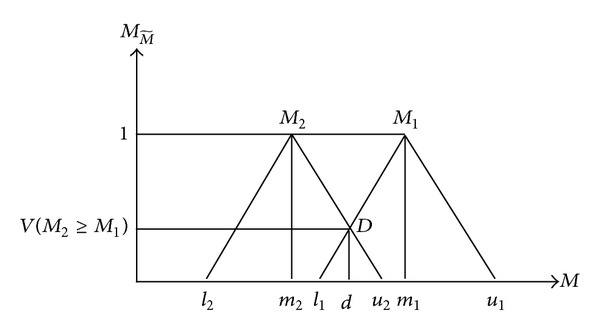
The intersection between *M*_1_ and *M*_2_.

**Table 1:** Criteria and subcriteria considered to evaluate EI.

Criteria	Subcriteria
C1: autonomy	C11: management style
	C12: having ownership
	C13: awareness of emerging technologies

C2: innovativeness	C21: technological innovation
	C22: product/market innovation

C3: risk taking	C31: venturing into the unknown business
	C32: heavy borrowing,
	C33: committing large portions of corporate assets in uncertain environments

C4: proactiveness	C41: being knowledgeable about current and future customers' preferences
	C42: developing plans
	C43: commitment to exploiting opportunities
	C44: anticipation of future demand

C5: competitiveness aggressiveness	C51: intensity of a firm's effort to outperform industry rivals
	C52: adopting unconventional tactics to challenge industry leaders

**Table 2:** Linguistic scales of importance.

Linguistic scale for importance	Triangular fuzzy scale	Triangular fuzzy reciprocal scale
Equal	(1, 1, 1)	(1, 1, 1)
Weak	(1/2, 1, 3/2)	(2/3, 1, 2)
Fairly strong	(3/2, 2, 5/2)	(2/5, 1/2, 2/3)
Very strong	(5/2, 3, 7/2)	(2/7, 1/3, 2/5)
Absolute	(7/2, 4, 9/2)	(2/9, 1/4, 2/7)

**Table 3:** Fuzzy pairwise comparisons of the main criteria.

Goal	Autonomy	Innovativeness	Risk taking	Proactiveness	Comp. aggr.
Autonomy	(1, 1, 1)	(2/5, 1/2, 2/3)	(1/2, 1, 3/2)	(2/5, 1/2, 2/3)	(2/5, 1/2, 2/3)
Innovativeness	(3/2, 2, 5/2)	(1, 1, 1)	(3/2, 2, 5/2)	(1/2, 1, 3/2)	(3/2, 2, 5/2)
Risk taking	(2/3, 1, 2)	(2/5, 1/2, 2/3)	(1, 1, 1)	(2/5, 1/2, 2/3)	(2/3, 1, 2)
Proactiveness	(3/2, 2, 5/2)	(2/3, 1, 2)	(3/2, 2, 5/2)	(1, 1, 1)	(1, 1, 1)
Comp. aggr.	(3/2, 2, 5/2)	(2/5, 1/2, 2/3)	(1/2, 1, 3/2)	(1, 1, 1)	(1, 1, 1)

**(a):** 

Autonomy	Management style	Having ownership	Awareness of emerging technologies
Management style	(1, 1, 1)	(1/2, 1, 3/2)	(2/7, 1/3, 2/5)
Having ownership	(2/3, 1, 2)	(1, 1, 1)	(2/5, 1/2, 2/3)
Awareness of emerging technologies	(5/2, 3, 7/2)	(3/2, 2, 5/2)	(1, 1, 1)

**(b):** 

Innovativeness	Technological innovation	Product/market innovation
Technological innovation	(1, 1, 1)	(1/2, 1, 3/2)
Product/market innovation	(2/3, 1, 2)	(1, 1, 1)

**(c):** 

Risk taking	Venturing into the unknown business	Heavy borrowing	Committing large portions
Venturing into the unknown business	(1, 1, 1)	(3/2, 2, 5/2)	(3/2, 2, 5/2)
Heavy borrowing	(2/5, 1/2, 2/3)	(1, 1, 1)	(2/3, 1, 2)
Committing large portions	(2/5, 1/2, 2/3)	(1/2, 1, 3/2)	(1, 1, 1)

**(d):** 

Proactiveness	Being knowledgeable	Developing plans	Commitment to exploiting opportunities	Anticipation of the future
Being knowledgeable	(1, 1, 1)	(3/2, 2, 5/2)	(1/2, 1, 3/2)	(1/2, 1, 3/2)
Developing plans	(2/5, 1/2, 2/3)	(1, 1, 1)	(2/5, 1/2, 2/3)	(2/5, 1/2, 2/3)
Commitment to exploiting opportunities	(2/3, 1, 2)	(3/2, 2, 5/2)	(1, 1, 1)	(1/2, 1, 3/2)
Anticipation of future	(2/3, 1, 2)	(3/2, 2, 5/2)	(2/3, 1, 2)	(1, 1, 1)

**(e):** 

Competitiveness aggressiveness	Intensity of a firm's effort	Adopting unconventional tactics
Intensity of a firm's effort	(1, 1, 1)	(1/2, 1, 3/2)
Adopting unconventional tactics	(2/3, 1, 2)	(1, 1, 1)

**Table 5:** Final weights of criteria and subcriteria using FAHP.

Criteria	Criteria weights	Subcriteria	Subcriteria weights	Local weights
C1: autonomy	0.08	C11: management style	0.44	0.035
		C12: having ownership	0.07	0.005
		C13: awareness of emerging	0.49	0.039

C2: innovativeness	0.33	C21: technological in.	0.5	0.165
		C22: product/market in.	0.5	0.165

C3: risk taking	0.1	C31: venturing into the unknown business	0.56	0.056
		C32: heavy borrowing	0.25	0.025
		C33: committing large portions	0.19	0.019

C4: proactiveness	0.29	C41: being knowledgeable	0.3	0.087
		C42: developing plans	0.1	0.029
		C43: commitment to exploiting opportunities	0.3	0.087
		C44: anticipation of future	0.3	0.087

C5: comp. aggr.	0.2	C51: intensity of a firm	0.5	0.1
		C52: adopting unconventional	0.5	0.1

**Table 6:** Linguistic variables for the criteria weights.

Very low (VL)	1
Low (L)	3
Medium (M)	5
High (H)	7
Very high (VH)	9
Intermediate value	2, 4, 6, 8

**Table 7:** The aggregated EI scores of the firms.

Firm	C1	C2	C3	C4	C5	C11	C12	C13	C21	C22	C31	C32	C33	C41	C42	C43	C44	C51	C52	EI
A1	5	7	5	5	5	3	5	**7**	7	6	5	1	3	7	3	6	5	6	7	0.606
A2	4	**8**	4	6	5	*2 *	5	6	6	5	4	1	2	8	4	7	5	5	5	0.599
A3	5	7	5	6	6	3	4	6	8	7	**7**	3	4	8	4	7	5	6	6	0.662
A4	6	7	5	**7**	7	4	6	7	8	**7**	6	2	5	7	5	**8**	6	7	7	0.71
A5	4	7	5	5	6	3	4	6	7	6	5	2	*2 *	6	5	6	4	6	6	0.6
A6	5	8	6	6	6	2	3	5	6	5	6	1	3	6	4	5	6	**7**	5	0.625
A7	6	6	4	6	7	4	4	7	6	5	7	2	5	7	4	6	**7**	6	5	0.617
A8	7	6	5	6	5	3	6	5	8	5	6	4	3	4	**6**	5	4	5	4	0.576
A9	3	5	5	4	7	**6**	3	6	5	4	4	4	5	5	3	4	5	6	5	0.511
A10	4	6	3	5	5	5	3	4	5	*3 *	4	1	4	5	4	6	5	6	7	0.518
A11	5	6	3	3	4	3	*2 *	5	6	5	5	3	2	3	2	4	4	4	4	0.457
A12	3	3	5	3	4	2	4	4	5	4	4	3	2	3	4	4	3	*2 *	4	0.371
A13	2	3	4	5	3	3	5	4	3	3	3	4	3	4	*2 *	5	*2 *	4	4	0.369
A14	*1 *	2	*3 *	4	2	3	4	4	4	3	*3 *	2	3	*2 *	2	4	3	4	*3 *	0.308
A15	3	5	4	4	4	4	5	3	6	4	4	**5**	4	3	3	*3 *	5	5	5	0.455
A16	4	5	4	3	*2 *	5	5	5	6	6	5	1	3	4	5	6	6	5	6	0.474
A17	4	7	5	6	6	3	4	6	7	5	5	2	2	6	5	6	4	5	6	0.61
A18	5	7	5	4	5	3	5	7	6	6	5	1	3	6	3	6	5	6	**7**	0.578
A19	3	7	5	6	5	3	3	6	**8**	6	7	3	3	8	4	7	4	6	6	0.629
A20	5	8	**6**	5	6	2	2	5	6	7	6	1	3	7	4	5	5	7	5	0.627
A21	7	6	4	6	5	4	**6**	5	8	7	6	5	4	4	6	5	4	5	5	0.598
A22	**7**	6	3	4	4	3	*2 *	5	5	5	5	5	2	3	2	4	5	4	5	0.484
A23	4	3	4	5	5	3	5	5	*3 *	3	5	4	3	4	3	5	*2 *	4	6	0.424
A24	5	5	6	3	4	4	6	*3 *	6	5	4	5	3	4	3	3	5	7	5	0.482
A25	6	8	5	6	**7**	3	5	6	7	5	5	2	3	6	5	6	5	5	6	0.642
A26	2	*2 *	4	4	3	3	4	5	6	3	3	3	4	2	2	5	3	4	7	0.375
A27	3	4	5	5	5	3	4	6	5	5	4	3	2	4	4	4	3	*2 *	5	0.454
A28	4	7	5	6	5	3	5	5	6	6	5	1	3	**8**	4	8	5	5	7	0.614
A29	5	7	4	5	7	4	5	7	6	4	7	*1 *	**5**	7	5	6	7	7	5	0.612
A30	3	4	6	*3 *	7	6	6	5	5	3	4	5	5	6	3	4	6	6	7	0.492

*W*	0.08	0.33	0.1	0.29	0.2	0.035	0.005	0.039	0.165	0.165	0.056	0.025	0.019	0.087	0.029	0.087	0.087	0.1	0.1	

**Table 8:** The values of *S* and *R* for all firms.

Firm	*S*	*R*	Firm	*S*	*R*	
A1	0.63	0.145	A16	1.13	0.29	*S*_j_* = 0.39 *R*_j_* = 0.072 *S*_*j*_^−^ = 1.78 *R*_*j*_^−^ = 0.33
A2	0.656	0.08	A17	0.647	0.082	
A3	0.39	0.072	A18	0.75	0.217	
A4	0.477	0.29	A19	0.526	0.082	
A5	0.659	0.145	A20	0.634	0.145	
A6	0.649	0.082	A21	0.635	0.109	
A7	0.577	0.109	A22	1.129	0.217	
A8	0.73	0.11	A23	1.344	0.274	
A9	1.17	0.217	A24	1.109	0.29	
A10	0.998	0.165	A25	0.496	0.082	
A11	1.25	0.29	A26	1.506	0.33	
A12	1.53	0.29	A27	1.181	0.219	
A13	1.53	0.274	A28	0.549	0.082	
A14	1.78	0.33	A29	0.627	0.145	
A15	1.219	0.217	A30	1.188	0.29	

**Table 9:** The ranking of the firms by *S*, *R*, and *Q* in an ascending order.

*Q*		*R*		*S*	
A3	0.0	A3	0.072	A3	0.39
A25	0.057	A25	0.082	A4	0.477
A19	0.063	A19	0.082	A25	0.496
A28	0.076	A28	0.082	A19	0.526
A2	0.11	A2	0.082	A28	0.549
A17	0.111	A17	0.082	A7	0.577
A6	0.112	A6	0.082	A29	0.627
A7	0.138	A7	0.109	A1	0.63
A21	0.159	A21	0.109	A20	0.634
A8	0.195	A8	0.11	A21	0.635
A29	0.226	A29	0.145	A17	0.647
A1	0.227	A5	0.145	A6	0.649
A20	0.228	A20	0.145	A2	0.656
A5	0.237	A1	0.145	A5	0.659
A10	0.398	A10	0.165	A8	0.73
A18	0.41	A18	0.217	A18	0.75
A4	0.453	A9	0.217	A10	0.998
A22	0.549	A22	0.217	A24	1.109
A9	0.561	A15	0.217	A22	1.129
A27	0.568	A27	0.219	A16	1.13
A15	0.579	A13	0.274	A9	1.17
A24	0.68	A23	0.274	A27	1.181
A16	0.688	A4	0.29	A30	1.188
A30	0.709	A30	0.29	A15	1.219
A11	0.731	A16	0.29	A11	1.25
A23	0.734	A24	0.29	A23	1.344
A13	0.801	A11	0.29	A26	1.506
A12	0.832	A12	0.29	A13	1.53
A26	0.901	A26	0.33	A12	1.53
A14	1	A14	0.33	A14	1.78

**Table 10:** Comparison of VIKOR and TOPSIS.

Firms	VIKOR_*Q*_	VIKOR_*R*_	VIKOR_*S*_	TOPSIS
A1	12	14	8	12
A2	5	5	13	10
A3	1	1	1	2
A4	17	23	2	1
A5	14	12	14	13
A6	7	7	12	6
A7	8	8	6	11
A8	10	10	15	14
A9	19	17	21	17
A10	15	15	17	16
A11	25	27	25	19
A12	28	28	29	28
A13	27	21	28	27
A14	30	30	30	30
A15	21	19	24	23
A16	23	25	20	22
A17	6	6	11	9
A18	16	16	16	15
A19	3	3	4	7
A20	13	13	9	3
A21	9	9	10	5
A22	18	18	19	18
A23	26	22	26	26
A24	22	26	18	20
A25	2	2	3	4
A26	29	29	27	29
A27	20	20	22	24
A28	4	4	5	8
A29	11	11	7	25
A30	24	24	23	21

## Appendix

Let *X*{*x*_1_, *x*_2_,…, *x*_*n*_}  be an object set, and let *U*{*u*_1_, *u*_2_,…, *u*_*n*_} be a goal set. According to the method of Chang (1992), each object is taken and extent analysis for each goal *g*_*i*_ is performed, respectively. Therefore, *m* extent analysis values for each object can be obtained, with the following signs

(A.1) $$\begin{array}{c} M_{g_{i}}^{1},M_{g_{i}}^{2},\ldots ,M_{g_{i}}^{m},\;i=1,2,\ldots ,n, \end{array}$$

where all the *M*_*g*_*i*__^*j*^  (*j* = 1,2,…, *m*) are TFNs. The steps of Chang's extent analysis can be given as in the following.

Step 16 . The value of fuzzy synthetic extent with respect to the *i*th object is defined as

(A.2) $$\begin{array}{c} S_{i}=\overset{m}{\underset{j=1}{\sum }}M_{gi}^{j}⊗{\left[\overset{n}{\underset{i=1\;}{\sum }}\overset{m}{\underset{j=1}{\sum }}M_{gi}^{j}\right]}^{-1}. \end{array}$$

To obtain [∑_*i*=1_^*n*^∑_*j*=1_^*m*^ *M*_*g*_*i*__^*j*^]^−1^, perform the fuzzy addition operation of *m* extent analysis values for a particular matrix such that

(A.3) $$\begin{array}{c} \overset{m}{\underset{j=1}{\sum }}M_{gi}^{j}=\left(\overset{m}{\underset{j=1}{\sum }}l_{i}\overset{m}{\underset{j=1}{\sum }}m_{i}\overset{m}{\underset{j=1}{\sum }}u_{i}\right), \end{array}$$

(A.4) $$\begin{array}{c} \overset{n}{\underset{i=1\;}{\sum }}\overset{m}{\underset{j=1}{\sum }}M_{gi}^{j}=\left(\overset{n}{\underset{j=1}{\sum }}l_{i}\overset{n}{\underset{j=1}{\sum }}m_{i}\overset{n}{\underset{j=1}{\sum }}u_{i}\right), \end{array}$$

and then compute the inverse of the vector in (A.4) such that

(A.5) $$\begin{array}{c} {\left[\overset{n}{\underset{i=1\;}{\sum }}\overset{m}{\underset{j=1}{\sum }}M_{g_{i}}^{j}\right]}^{-1}=\left(\frac{1}{\sum_{i=1}^{n}u_{i}},\frac{1}{\sum_{i=1}^{n}m_{i}},\frac{1}{\sum_{i=1}^{n}l_{i}}\right). \end{array}$$

Step 17 . The degree of possibility of *M*_2_ = (*l*_2_, *m*_2_ *u*_2_) ≥ *M*_1_ = (*l*_2_, *m*_2_ *u*_2_) is defined as

(A.6) V(M2≥M1)=⌊min⁡(μM1(x),μM2(y))⌋y≥xSUP,

which can be equivalently expressed as follows:

(A.7) V(M2≥M1)=hgt(M1∩M2)=μM2(d)={1if  m2≥m10if  l1≥u2l1−u2(m2−u2)−(m1−l1)otherwise,

where *d* is the ordinate of the highest intersection point *D* between *μ*_*M*_1__and *μ*_*M*_2__ (see Figure 3).

To compare *M*_1_ and *M*_2_, we need both of the values of *V*(*M*_1_ ≥ *M*_2_) and  *V*(*M*_2_ ≥ *M*_1_).

Step 18 . The degree possibility for a convex fuzzy number to be greater than *k* convex fuzzy numbers *M*_*i*_  (*i* = 1,2,…, *k*) can be defined by

(A.8) V(M≥M1,M2,…,MK)  =V[(M≥M1)  and  (M≥M2)  and…and(M≥MK)]  =min⁡⁡V(M≥Mi), i=1,2,3,…,k.

Assume that

(A.9) $$\begin{array}{c} d^{\prime }\left(A_{i}\right)=\min V\left({S_{i}\geq S}_{k}\right), \end{array}$$

for *k* = 1, 2,…*n*, *k* ≠ *i*. Then the weight vector is given by

(A.10) $$\begin{array}{c} W^{\prime }={\left(d^{\prime }\left(A_{1}\right),d^{\prime }\left(A_{2}\right),\ldots ,d^{\prime }\left(A_{n}\right)\right)}^{T}, \end{array}$$

where *A*_*i*_  (*i* = 1,2,…, *n*) are *n* elements.

Step 19 . Via normalization, the normalized weight vectors are given by

(A.11) $$\begin{array}{c} W={\left(d\left(A_{1}\right),d\left(A_{1}\right),\ldots ,d\left(A_{1}\right)\right)}^{T}, \end{array}$$

where *W* is a nonfuzzy number.
